# CNS efficacy of afatinib as first-line treatment in advanced non-small cell lung cancer patients with EGFR mutations

**DOI:** 10.3389/fonc.2023.1094195

**Published:** 2023-02-23

**Authors:** Liping Kang, Jianliang Mai, Weiting Liang, Qihua Zou, Caiwen Huang, Yongbin Lin, Ying Liang

**Affiliations:** ^1^ Department of Medical Oncology, State Key Laboratory of Oncology in South China, Collaborative Innovation Center for Cancer Medicine, Sun Yat-sen University Cancer Center, Guangzhou, China; ^2^ State Key Laboratory of Oncology in South China, Collaborative Innovation Center for Cancer Medicine, Sun Yat-sen University Cancer Center, Guangzhou, China; ^3^ Department of Medical Oncology, Cancer Hospital Chinese Academy of Medical Sciences, Shenzhen Center, Shenzhen, China; ^4^ Department of Thoracic Surgery, State Key Laboratory of Oncology in South China, Collaborative Innovation Center for Cancer Medicine, Sun Yat-sen University Cancer Center, Guangzhou, China

**Keywords:** NSCLC, afatinib, brain metastases, CNS efficacy, uncommon EGFR mutations

## Abstract

**Background:**

Afatinib is a potent, irreversible second-generation epidermal growth factor receptor (EGFR) tyrosine kinase inhibitor which has demonstrated efficacy in advanced non-small cell lung cancer (NSCLC) patients harboring either common or uncommon EGFR mutations. However, data on its activity against brain metastases are limited. This study aimed to retrospectively evaluate the efficacy and safety of afatinib as first-line treatment for EGFR-mutant NSCLC patients with brain metastases.

**Methods:**

Treatment-naive advanced NSCLC patients harboring EGFR mutations and brain metastases treated with afatinib were retrospectively reviewed to assess the central nervous system (CNS) efficacy and also the systematic benefits.

**Results:**

Totally 43 patients with measurable or non-measurable brain metastases were enrolled in the CNS full analysis (cFAS) set. Among them, 23 patients with measurable brain metastases were included in the CNS evaluable for response (cEFR) set. The CNS ORR was 48.8% (95% CI, 33.3 - 64.5%) in the cFAS set and 82.6% (95% CI, 61.2 - 95.0%) in the cEFR set, respectively. CNS mDoR was 8.9 months (95% CI, 4.7 - 13.1 months) and CNS mPFS was 12.7 months (95% CI, 6.9 - 18.5 months) in the cFAS set. In the subgroup analysis stratified by EGFR mutation types, CNS ORR of cEFR set in the common mutation cohort was 100% (95% CI, 75.3 - 100%) and 60% (95% CI, 26.2 - 87.8%) in the uncommon mutation cohort (*p* = 0.024); CNS ORR of cFAS set was 57.7% (95% CI, 36.9 - 76.6%) and 35.3% (95% CI, 14.2 - 61.7%), respectively (*p* = 0.151). CNS mPFS was 14.4 months in patients with common mutations and 6.1 months in patients with uncommon mutations (hazard ratio, 0.47; 95% CI, 0.22 - 1.00; *p* = 0.045). Patients with common mutations showed a significantly lower cumulative incidence of CNS failure than uncommon mutation cohort (*p* = 0.0026). Most of patients experienced grade 1/2 treatment-related adverse events.

**Conclusions:**

First-line afatinib demonstrated encouraging efficacy on brain metastases in NSCLC patients harboring either common or major uncommon EGFR mutations in a real-world setting, with manageable toxicities. Patients with common mutations showed better CNS outcomes than those with uncommon mutations.

## 1 Introduction

Brain metastases (BMs) occur in approximately 30% to 50% of non-small cell lung cancer (NSCLC) patients during the whole course of the disease, indicating poor prognosis and great challenges for treatment (1–3). Epidermal growth factor receptor (EGFR) mutation is one of the most pervasive oncogenic driver mutations in NSCLC, which is found in approximately 15% to 20% of Caucasian patients and 30% to 50% of Asian patients (4–6). The two most common EGFR mutations, exon 19 deletion (19 del) and exon 21 Leu858Arg (L858R) mutation account for approximately 80% to 90% of this oncogenic alteration, while uncommon EGFR mutations are estimated as approximately 10% to 20% (7–10). Patients with EGFR mutations are more prone to BMs than those with wild-type (11). Traditionally, the mainstream treatment options for NSCLC patients with BMs include surgical resection, stereotactic radiosurgery (SRS), and whole-brain radiotherapy (WBRT). However, these strategies may lead to radiation necrosis and significant compromises of loss of neurocognitive function (12–14). In the past two decades, the remarkable improvements by the molecular-targeted therapies have been seen in patients with NSCLC driven by oncogenic alterations, especially in EGFR tyrosine kinase inhibitors (TKIs). On the basis of the favorable results of prospective randomized trials, EGFR TKIs are now recommended as a standard first-line treatment replacing conventional platinum-based chemotherapy for patients with EGFR-mutated NSCLC (15–20). As a result of prolonging survival afforded by EGFR TKIs coupled with improvements of neuroimaging technology, patients seemed more inclined to develop BMs, with a 3-year cumulative risk of developing BMs increased to roughly 47% over the course of disease (21). Data on improved central nervous system (CNS) efficacy and manageable toxicities by some EGFR TKIs have also been reported previously (19, 20, 22–27). Given these, it’s crucial to further explore the CNS efficacy of EGFR TKIs and optimize the first-line treatment and subsequent strategies for patients with BMs under the consideration of both overall survival benefit and patient quality of life.

Afatinib is a second-generation, irreversible ErbB family blocker that selectively blocks signals from ErbB family receptors (EGFR [ErbB1], HER2 [ErbB2], and ErbB4) and transphosphorylation of ErbB3, which cause a more sustained and wider-spectrum activity against EGFR mutations in contrast to reversible first-generation EGFR TKIs (erlotinib and gefitinib). Owing to its favorable efficacy in LUX-Lung series, afatinib was approved of the first-line treatment for NSCLC patients with EGFR mutations. In a combined analysis of LUX-lung 3 and 6 for common EGFR mutations and BMs (n = 48), afatinib demonstrated significant clinical activity against BMs with a CNS objective response rate (ORR) of 72.9% and median CNS progression-free survival (PFS) of 8.2 months (23). It also showed favorable CNS efficacy and survival outcomes in the real-world studies, irrespective of the EGFR mutation types (28, 29). And based on a series of reported findings mainly focus on common mutations, afatinib appeared to show a trend toward superiority over chemotherapy and first-generation EGFR TKIs in terms of CNS PFS, CNS ORR and cumulative incidence risk of CNS failure in patients with BMs (19, 22–25). Additionally, due to its significant clinical benefits in uncommon EGFR mutations such as G719X, S768I, L861Q, and some compound mutations (defined as ≥2 EGFR mutations and at least one uncommon EGFR mutation), afatinib is currently the only EGFR TKI approved for advanced NSCLC patients with G719X/L861Q/S768I (30). However, there were very few reports on the activity of afatinib for BMs in uncommon EGFR-mutant NSCLC patients.

There is still an unmet need to comprehensively assess the CNS efficacy of afatinib, especially in patients harboring uncommon mutations in the real-world setting. We conducted this study to explore its activity and tolerability in EGFR-TKI-naive patients with baseline BMs, expecting to help guide therapeutic selections of appropriate EGFR TKIs and thus to provide guidance for clinical practice.

## 2 Methods

### 2.1 Patients

EGFR-mutant NSCLC patients with BMs who received afatinib (30 mg or 40 mg, orally, once daily) as first-line treatment at Sun Yat-Sen University Cancer Center between March 2018 and January 2022 were retrospectively reviewed in this study. Patients received contrast computed tomography (CT) scans and contrast magnetic resonance imaging (MRI) at baseline and reviewed every 8 weeks from the start of afatinib until treatment discontinuation. Clinical and imaging data of eligible patients were extracted from the electronic medical records for response evaluation. This retrospective study was approved by the Institutional Review Board of Sun Yat-Sen University Cancer Center and conducted in accordance with the Declaration of Helsinki.

#### 2.1.1 Inclusion and exclusion criteria

Patients who met the inclusion and exclusion criteria were eligible for evaluation in this retrospective study. The inclusion criteria details were as follows (1): at least 18 years of age (2), pathologically confirmed NSCLC, (3) contrast MRI-detected BMs at baseline, (4) BMs without prior radiotherapy including asymptomatic BMs or BMs with focal neurological symptoms but no need for steroids, (5) laboratory-confirmed EGFR mutations detected by real-time PCR, Sanger sequencing, amplification-refractory mutation system (ARMS)-polymerase chain reaction (PCR) or next-generation sequencing, (6) at least one measurable extracranial lesion, defined as ≥10 mm, (7) an Eastern Cooperative Oncology Group (ECOG) performance status of 0 - 2, (8) no previous treatment with antineoplastic agents after initial diagnosis. The exclusion criteria were: (1) *de novo* EGFR T790M mutation and EGFR exon 20 insertion, (2) accompanied by other malignant tumors, (3) a combination with other anti-tumor agents.

### 2.2 Assessment

Treatment response was assessed by Response Evaluation Criteria in Solid Tumors, version 1.1 (RECIST 1.1) for both intracranial lesions and extracranial lesions. Measurable lesions were defined as target lesions (TLs)and non-measurable lesions as nontarget lesions (NTLs). Patients with measurable and/or non-measurable brain lesions at baseline were included in the CNS full analysis (cFAS) set. Patients with at least one measurable brain lesion at baseline were included in the CNS evaluable for response (cEFR) set. Besides that, subgroup analysis was made according to EGFR mutation subtypes. Severity of adverse events were recorded on the basis of Common Terminology Criteria for Adverse Events, version 5.0 (CTCAE 5.0).

### 2.3 Statistical analysis

CNS ORR, CNS disease control rate (DCR), CNS duration of response (DoR), CNS PFS, CNS time to response (TTR), cumulative incidence of CNS failure and best percentage change from baseline in TL size were recorded to evaluate the CNS response. CNS ORR was defined as the percentage of patients who achieved a best CNS response of complete response (CR) or partial response (PR). CNS DCR was defined as the proportion of patients with a CR or PR or stable disease (SD) in brain lesions. CNS DoR was defined as the time from first documentation of intracranial CR or PR until the time of progression (including intracranial progressive disease [PD] or extracranial PD) or death of any reason, whichever came first. CNS PFS was defined as the time from the first dose of afatinib until the time of progression (including intracranial PD or extracranial PD) or death of any reason, whichever came first. And CNS TTR was defined as the time from the first dose of afatinib to the time when the intracranial CR or PR to afatinib was first evaluated. The ORR and DCR were calculated with exact Clopper-Pearson 95% confidence intervals (CIs) based on the exact binomial distribution, and compared by chi-square test or Fisher’s exact test. CNS DoR, PFS, and TTR were estimated by the Kaplan-Meier method with corresponding 95% CIs, and compared by log-rank test. Besides, a Cox proportional hazards model was applied to estimate HRs and 95% CIs with significance set at *p <*0.05 level. A competing risk analysis estimating the cumulative incidence for the event of interest (CNS progression) in the presence of competing risk event (non-CNS progression) was performed using a semiparametric Fine–Gray regression model. All the *p* values reported in the analysis were two-sided, and a *p <*0.05 level was considered statistically significant in the tests. And all statistical analyses were calculated using SPSS (version 26.0) except for the competing risks analysis, which were calculated with R software (version 4.1.2), and plots were executed using R software (version 4.1.2).

## 3 Results

### 3.1 Patient characteristics

By the data cut-off date as January 20, 2022, a total of 43 EGFR-mutant NSCLC patients with BMs at first diagnosis were enrolled in this retrospective analysis. The detailed baseline demographics and clinical characteristics of patients are presented in **Table 1**. Among these patients, 26 (60.5%) were male and 17 (39.5%) were female. The median age was 57 years (range, 37 - 79 years). All of them were Asians (Chinese), and most of them were adenocarcinoma (42 of 43, 97.7%) and nonsmokers (27/43, 62.8%). All patients were diagnosed with brain parenchymal metastases, none had leptomeningeal metastases. 4 (9.3%) patients had mild baseline CNS symptoms associated with brain metastases, including headache in 3 (7.0%) patients and dizziness in 1 (2.3%) patient. EGFR mutation status was confirmed by molecular pathology, with tumor biopsy tissue samples used in 35 (81.4%) patients, blood samples in 6 (14.0%) patients and pleural effusions samples in 2 (4.7%) patients. 26 (60.5%) patients were reported to have common EGFR mutations (16 [37.2%] were exon 19 deletions, 10 [23.3%] were exon 21 Leu858Arg), and 17 (39.5%) were reported to have uncommon mutations (3 [7.0%] were G719X, 2 [4.7%] were L861Q, 1 [2.3%] were S768I, and 11[25.6%] were compound mutations).

**Table 1:** Baseline demographics and clinical characteristics of patients.

Characteristic	Patients,n (%) (n = 43)
Gender	
Male	26 (60.5)
Female	17 (39.5)
Age,years	
Median age, years (range)	57 (37-79)
< 65	31 (72.1)
≥ 65	12 (27.9)
Race	
Asians	43 (100.0)
Smoking status	
Never	27 (62.8)
Current or former	16 (37.2)
ECOG PS	
0-1	40 (93.0)
2	3 (7.0)
Histologic type	
Adenocarcinoma	42 (97.7)
Adenosquamous carcinoma	1 (2.3)
EGFR mutation type	
Exon 19 del	16 (37.2)
L858R	10 (23.3)
Uncommon mutation	17 (39.5)
G719X	3 (7.0)
L861Q	2 (4.7)
S768I	1 (2.3)
Compound mutation	11 (25.6)
G719X+Exon 19 del	1 (2.3)
G719X+L861Q	2 (4.7)
G719X+E709X	2 (4.7)
G719X+S768I	2 (4.7)
G719X+V769M	1 (2.3)
S768I+L858R	1 (2.3)
E709X+L858R	1 (2.3)
L861Q+L833W	1 (2.3)
Patients with measurable brain lesions	
Yes	23 (53.5)
No	20 (46.5)
Number of brain lesions, n (%)	
1	8 (18.6)
2-5	12 (27.9)
>5	23 (53.5)
Site of distant metastasis	
Contralateral lung	24 (55.8)
Liver	12 (27.9)
Pleura	17 (39.5)
Pancreas	2 (4.7)
Bone	32 (74.4)
Adrenal gland	11 (25.6)
Abdominal/Pelvic cavity	5 (11.6)
Starting dose	
30mg	26 (60.5)
40mg	17 (39.5)

ECOG, Eastern Cooperative Oncology Group; PS, performance status; EGFR, epidermal growth factor receptor.

### 3.2 Treatment

Afatinib starting dose of 30 mg once daily was given to 26 patients and 40 mg once daily given to 17 patients as oncologist’s option based on the integrative consideration of individual risk-benefit profile according to individual conditions such as age, weight and comorbidities, etc. Generally, older patients (≥70 years) and those with lower body weight (<50 kg) would more trend to start at 30mg once daily. All patients had never received prior EGFR TKIs or cytotoxic drugs for anti-cancer treatment.

### 3.3 Efficacy

#### 3.3.1 CNS efficacy

Totally, 43 patients were eligible for CNS response evaluation as the cFAS set, of which 23 were included in the cEFR set.

**Figure 1:**
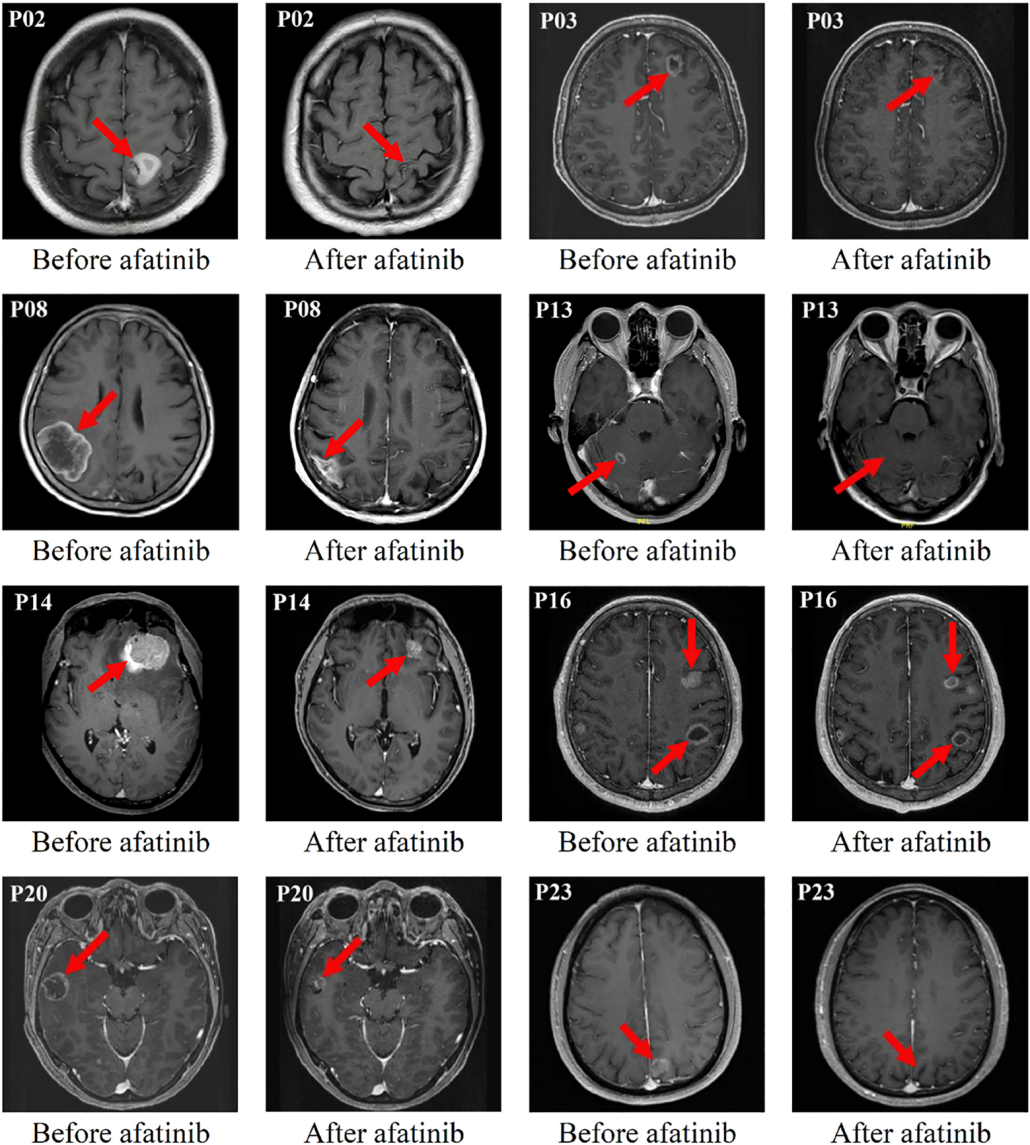
Eight typical examples of brain contrast MRI radiological changes in patients with measurable brain lesions (i.e. The red arrow points).

**Figure 2:**
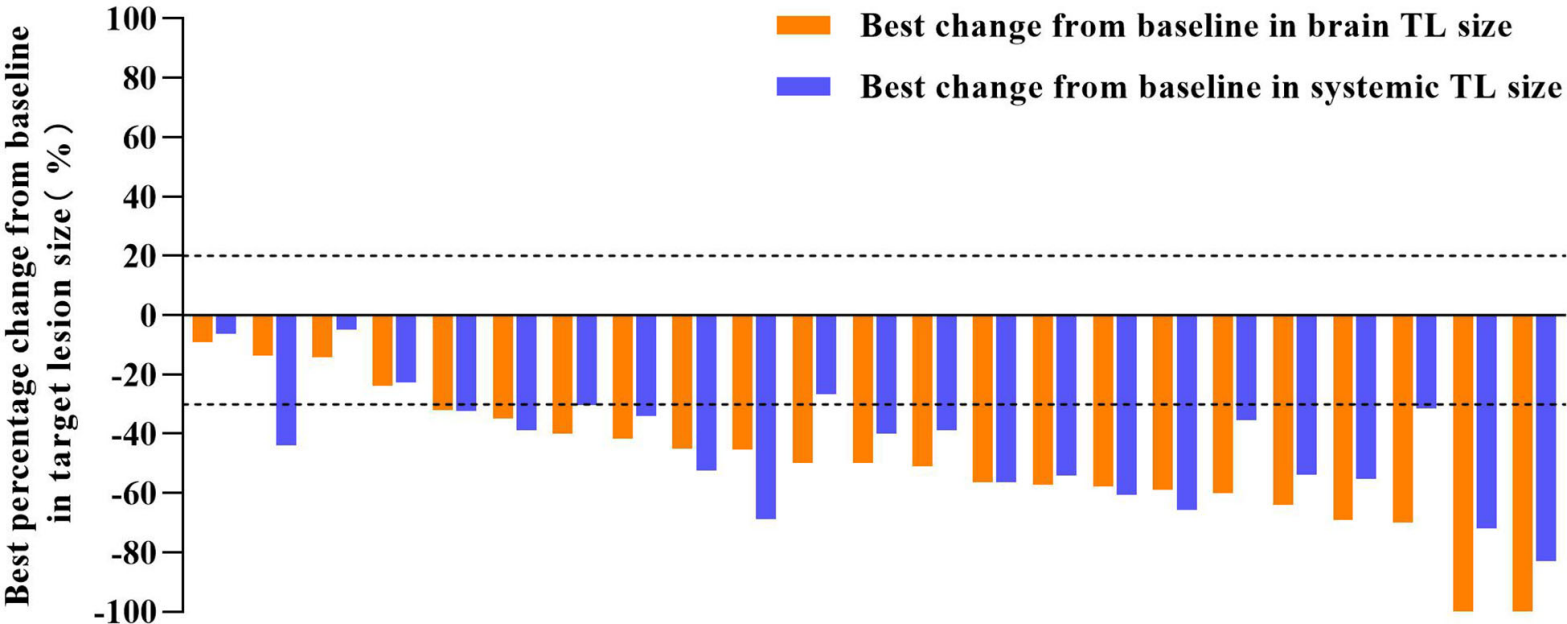
Tumor shrinkage in target lesion (TL) size of cEFR set. The median best percentage change from baseline in the sum of brain TL size was -53.7% (range, -100.0% to -9.1%). The median best percentage change from baseline in the sum of systemic TL size was -47.7% (range, -83.0% to -6.3%).

In the cEFR set, the CNS ORR was 82.6% (95% CI, 61.2 - 95.0%) and the CNS DCR was 100% (95% CI, 85.2 - 100%), with 2 CR (8.7%), 17 PR (73.9%), and 4 SD (17.4%) (**Table 2** and **Figure 1**). The CNS mPFS was 12.7 months (95% CI, 8.7 - 16.7 months). The CNS mDoR was 8.9 months (95% CI, 5.0 - 12.8 months). The median best percentage change from baseline in the sum of CNS TL size was -53.7% (range, -100.0% to -9.1%) (**Figure 2**).

**Table 2:** CNS activity of afatinib in patients with brain metastases.

Analysis Set/Response	cEFR (n = 23)	cFAS (n=43)
CNS Best overall response, n (%)		
CR	2 (8.7)	4 (9.3)
PR	17 (73.9)	17 (39.5)
SD or non-CR/non-PD*	4 (17.4)	22 (51.2)
PD	0 (0.0)	0 (0.0)
CNS ORR,% (95% CI)	82.6 (61.2-95.0)	48.8 (33.3-64.5)
CNS DCR, % (95% CI)	100.0 (85.2-100.0)	100.0 (91.8-100.0)
CNS DoR		
Median, months (95% CI)	8.9 (5.0-12.8)	8.9 (4.7-13.1)
CNS PFS		
Median, months (95% CI)	12.7 (8.7-16.7)	12.7 (6.9-18.5)
Follow-up time		
Median, months (95% CI)	23.0 (5.6-40.4)	16.7 (10.9-22.5)
CNS TTR,month		
Median, months (95% CI)	1.6 (1.3-2.0)	1.6 (1.3-2.0)
Estimated % remaining in response (95% CI)		
At 3 months	83.5 (68.0-100.0)	85.2 (70.9-100.0)
At 6 months	64.2 (44.8-92.2)	62.5 (43.8-89.1)
At 9 months	47.6 (27.2-83.2)	48.2 (28.9-80.3)
CNS PFS, % (95% CI)		
Progression free at 6 months	85.5 (71.6-100.0)	74.7 (62.2-89.6)
Progression free at 12 months	55.3 (35.9-85.1)	51.2 (37.1-70.7)

CNS, central nervous system; cEFR, CNS evaluable for response; cFAS, CNS full analysis; CR, complete response; PR, partial response; SD, stable disease; PD, progressive disease; ORR, objective response rate; DCR, disease control rate; CI, confidence interval; DoR, duration of response; PFS, progression-free survival; TTR, time to response.

*CNS response in patients with nontarget lesions only was classified as CR, non-CR, progressive disease (PD), or non-PD, but neither PR nor SD. Stable disease includes non-CR, non-PD in patients with nontarget lesions.

**Figure 3:**
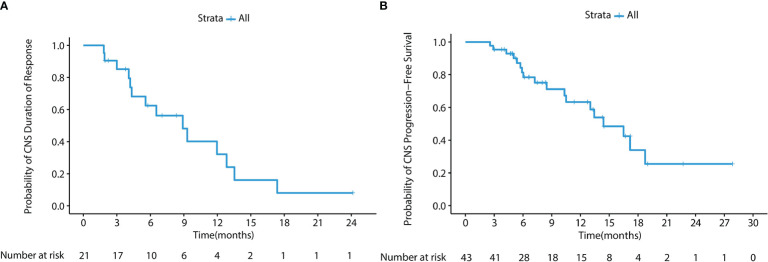
**(A)** Kaplan-Meier survival curve of CNS DoR in the cFAS set. The CNS mDoR was 8.9 months (95% CI, 4.7 - 13.1 months). **(B)** Kaplan-Meier survival curve of CNS PFS in the cFAS set. The CNS mPFS was 12.7 months (95% CI, 6.9 - 18.5 months).

In the cFAS set, the CNS ORR was 48.8% (95% CI, 33.3 - 64.5%) and the CNS DCR was 100% (95% CI, 91.8 - 100%), with 4 CR (9.3%), 17 PR (39.5%), and 22 SD (51.2%) (**Table 2**). The CNS mPFS was 12.7 months (95% CI, 6.9 - 18.5 months), with a 6-month CNS PFS rate of 74.7% (95% CI, 62.2 - 89.6%) and a 1-year CNS PFS rate of 51.2% (95% CI, 37.1 - 70.7%). The CNS mDoR was 8.9 months (95% CI, 4.7 - 13.1 months), with the estimated proportion of patients remaining in CNS response at 3, 6, and 9 months of 85.2%, 62.5% and 48.2%, respectively (**Figures 3A, B**). The CNS mTTR was 1.6 months (95% CI, 1.3 - 2.0 months), which were the same as the cEFR set. The baseline neurological symptoms in 4 patients were obviously improved after starting afatinib.

**Figure 4:**
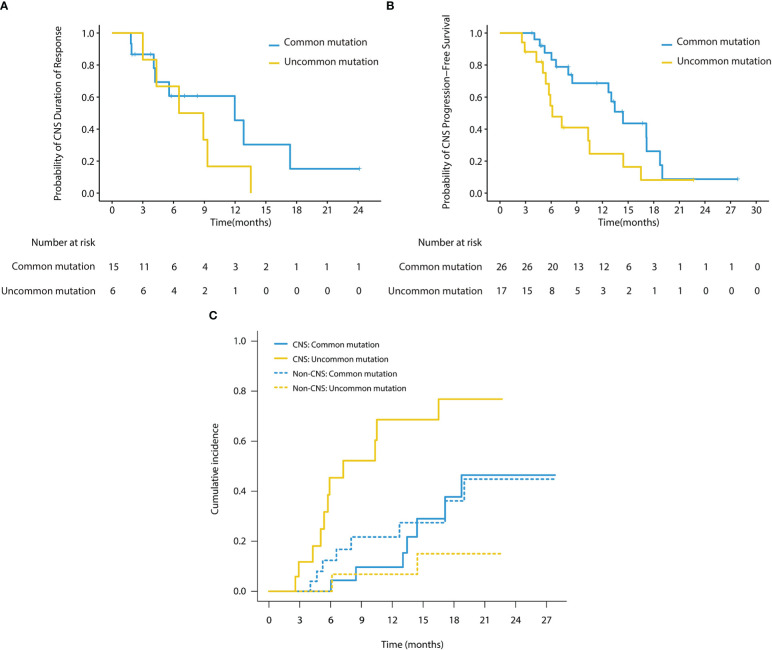
**(A)** Kaplan-Meier survival curves of CNS DoR in subgroup analysis (cFAS). The CNS mDoR was 12.0 months (95% CI, 4.1 - 19.9 months) in patients with common mutations and 6.5 months (95% CI, 1.1 - 12.0 months) in patients with uncommon mutations (HR, 0.57; 95% CI, 0.19 - 1.71; *p* = 0.31). **(B)** Kaplan-Meier survival curves of CNS PFS in subgroup analysis (cFAS). The CNS mPFS was 14.4 months (95% CI, 12.0 - 16.8 months) in patients with common mutations and 6.1 months (95% CI, 4.3 - 8.0 months) in patients with uncommon mutations (HR, 0.47; 95% CI, 0.22 - 1.00; *p* = 0.045). **(C)** Cumulative incidence of CNS failure in patients with baseline brain metastases. Patients with common mutations showed a significantly lower cumulative incidence of CNS failure compared with those with uncommon mutations (*p* = 0.0026), with the estimated probability of CNS progression at 12 months of 9.7% and 68.6%, respectively.

In the subgroup analysis stratified by EGFR mutation subtypes, as shown in **Table 3**, patients with common mutations (n = 13) achieved a significantly higher CNS ORR than those with uncommon mutations (n = 10) in the cEFR set (13 of 13 [100.0%] vs 6 of 10 [60.0%]; *p* = 0.024), as well as numerically higher CNS ORR in the cFAS set, though without statistical significance (15 of 26 [57.7%] vs 6 of 17 [35.3%]; *p* = 0.151). CNS mPFS was significantly longer in the common mutation group than the uncommon mutation group (14.4 months [95% CI, 12.0 - 16.8 months] vs 6.1 months [95% CI, 4.3 - 8.0 months]; HR 0.47; 95% CI, 0.22 - 1.00; *p* = 0.045) (**Figures 4A, B**). There were no significant differences in mDoR (12.0 months [95% CI, 4.1 - 19.9 months] vs 6.5 months [95% CI, 1.1 - 12.0 months]; HR, 0.57; 95% CI, 0.19 - 1.71; *p* = 0.310) and mTTR (2.0 months [95% CI, 0.6 - 3.3 months] vs 1.0 months [95% CI, 0.2 - 1.8 months]; HR, 0.43; 95% CI, 0.16 - 1.20; *p* = 0.097) in both groups. In the competing risk analysis for cumulative incidence of CNS failure, patients with common mutations showed a significantly lower cumulative incidence of CNS failure compared with those with uncommon mutations (*p* = 0.0026), with the estimated probability of CNS progression at 12 months of 9.7% and 68.6%, respectively (**Figure 4C**). Briefly, the efficacy outcome in the common mutation group was generally better than the uncommon group.

**Table 3:** CNS activity of afatinib in patients harboring common mutations or uncommon mutations.

Analysis Set/Response	cEFR		cFAS	
	Uncommon mutation (n =10)	Common mutation (n =13)	Uncommon mutation (n=17)	Common mutation (n=26)
CNS Best overall response, n (%)				
CR	1 (10.0)	1 (7.7)	1 (5.9)	3 (11.5)
PR	5 (50.0)	12 (92.3)	5 (29.4)	12 (46.2)
SD or non-CR/non-PD	4 (40.0)	0 (0.0)	11 (64.7)	11 (42.3)
PD	0 (0.0)	0 (0.0)	0 (0.0)	0 (0.0)
CNS ORR, % (95% CI)	60.0 (26.2-87.8)	100.0 (75.3-100.0)	35.3 (14.2-61.7)	57.7 (36.9-76.6)
CNS DCR, % (95% CI)	100.0 (69.2-100.0)	100.0 (75.3-100.0)	100.0 (80.5-100.0)	100.0 (86.8-100.0)
CNS DoR				
Median, months (95% CI)			6.5 (1.1-12.0)	12.0 (4.1-19.9)
CNS PFS				
Median, months (95% CI)			6.1 (4.3-8.0)	14.4 (12.0-16.8)
TTR,month				
Median, months (95% CI)			1.0 (0.2-1.8)	2.0 (0.6-3.3)
Estimated % remaining in response (95% CI)				
At 3 months			83.3 (58.3-100.0)	86.7 (71.1-100.0)
At 6 months			66.7 (37.9-100.0)	60.7 (38.6-95.3)
At 9 months			33.3 (10.8-100.0)	45.5 (22.1-94.0)
PFS, % (95% CI)				
Progression free at 6 months			54.6 (34.5-86.5)	87.6 (75.4-100.0)
Progression free at 12 months			24.6 (9.6-62.9)	68.7 (51.7-91.2)
12-month cumulative incidence rate of CNS failure			68.6%	9.7%

#### 3.3.2 Systemic efficacy

Forty-three people with measurable TLs were eligible for systemic response evaluation. ORR was 79.1% (95% CI, 64.0 - 90.0%) and DCR was 100% (95% CI 91.8 - 100.0%), with 34 PR (79.1%) and 9 SD (20.9%) (**Table 4**). The median best percentage change from baseline in systemic TL size was -47.7% (range, -83.0% to -6.3%). In subgroup analysis, ORR was 92.3% (95% CI, 74.9 - 99.1%) in patients with common mutations and 58.8% (95% CI, 32.9 - 81.6%) in patients with uncommon mutations (*p* = 0.018). DCRs of both subgroups were 100%.

**Table 4:** Systemic activity of afatinib in patients with brain metastases.

Analysis Set/Response	Uncommon mutation (n = 17)	Common mutation (n = 26)	All patients (n = 43)
Best overall response, n (%)			
CR	0 (0.0)	0 (0.0)	0 (0.0)
PR	10 (58.8)	24 (92.3)	34 (79.1)
SD	7 (41.2)	2 (7.7)	9 (20.9)
PD	0 (0.0)	0 (0.0)	0 (0.0)
ORR, % (95% CI)	58.8 (32.9-81.6)	92.3 (74.9-99.1)	79.1 (64.0-90.0)
DCR, % (95% CI)	100.0 (80.5-100.0)	100.0 (86.8-100.0)	100 (91.8-100.0)

### 3.4 Safety

**Table 5** summarizes the most common treatment-related adverse events (TRAEs). The most common TRAEs (any grade) were skin rash or acne (37 of 43,86.0%), diarrhea (35 of 43, 81.4%), stomatitis or mucositis (30 of 43, 69.8%), and paronychia (25 of 43, 58.1%). Forty (93.0%) patients experienced at least one grade 1 - 2 TRAEs. Grade 3 TRAEs were reported in four (9.3%) patients, including one (2.3%) with rash or acne, two (4.7%) with diarrhea, one (2.3%) with thrombocytopenia. Grade 4 TRAEs or treatment related death were not seen. Among all 43 patients, only one patient permanently discontinued afatinib treatment due to Grade 3 rash. Four patients experienced temporary afatinib discontinuation for approximately one week due to intolerable TRAEs, after which the afatinib dose was reduced from 40mg to 30mg once daily to continue treatment. Five patients were tolerated well thus had afatinib dose escalation from 30mg to 40mg once daily for better clinical benefits. Overall, no unexpected TRAEs of afatinib were observed and most AEs were manageable and tolerable.

**Table 5:** TRAEs of afatinib in patients with brain metastases.

TRAEs (n = 43)	All grades, n (%)	Grades 3–4, n (%)
Any TRAE	40 (93.0)	4 (9.3)
Rash or acne	37 (86.0)	1 (2.3)
Diarrhea	35 (81.4)	2 (4.7)
Stomatitis or mucositis	30 (69.8)	0 (0.0)
Paronychia	25 (58.1)	0 (0.0)
Pruritus	4 (9.3)	0 (0.0)
Decreased appetite	6 (14.0)	0 (0.0)
Vomiting	0 (0.0)	0 (0.0)
Nausea	2 (4.7)	0 (0.0)
Constipation	2 (4.7)	0 (0.0)
Fatigue	3 (7.0)	0 (0.0)
Alopecia	0 (0.0)	0 (0.0)
Increased ALT/AST	6 (14.0)	0 (0.0)
Anaemia	0 (0.0)	0 (0.0)
Leukopenia	2 (4.7)	0 (0.0)
Neutropenia	0 (0.0)	0 (0.0)
Thrombocytopenia	0 (0.0)	1 (2.3)

TRAEs, treatment-related adverse events.

### 3.5 Follow-up

At data cut-off, 28 patients experienced disease progressions, with 13 intracranial PD only, 10 extracranial PD only, 5 both intracranial and extracranial PD. All patients with disease progressions discontinued afatinib treatment. Among the thirteen patients with intracranial progressions only, two patients were lost to follow-up, eight had genetic reassessment, of which acquired EGFR T790M-positive status was confirmed by blood sample in one patient and the same EGFR mutations remained detectable in the other seven patients, with blood samples used in five patients, cerebrospinal fluid sample in one patient and pleural effusion sample in one patient. Additionally, the remaining three patients without genetic reassessment were subsequently treated with radiotherapy for brain metastatic lesions, one had WBRT, one had stereotactic body radiotherapy (SBRT) and one had three-dimensional conformal radiotherapy (3D-CRT). The patient with EGFR T790M mutation switched to osimertinib. Other patients were switched to chemotherapy (with/without bevacizumab).

## 4 Discussion

To the best of our knowledge, afatinib is an irreversible second-generation EGFR TKI that has been approved for the first-line treatment for patients with EGFR-mutated NSCLC. Currently, evidence of its efficacy for initial treatment of BMs is rarely reported, especially in those harboring uncommon mutations. This retrospective study provided encouraging CNS ORR, CNS mPFS and other survival data to support that first-line afatinib was also favorable to control BMs in EGFR-positive NSCLC patients, with an acceptable safety profile, even in those with certain uncommon EGFR mutations.

Our data further strengthened the clinical benefits of afatinib to BMs. The efficacy of afatinib on BMs in the cEFR set as demonstrated by both CNS ORR (82.6%) and CNS mPFS (12.7 months) was relatively consistent with the previous findings (22–25, 28, 29). More specifically, in both of the cEFR and cFAS sets, CNS mPFS was longer than that reported in the combined analysis of LUX-lung 3 and 6 as well as LUX-lung 7, which is 8.2 months and 7.2 months in common EGFR-mutant patients, respectively (19, 23). A probable explanation for this could be the inherent limitation of this single-center retrospective analysis, in which selection bias was inevitable. Long-term maintenance of afatinib and more effective management of TRAEs may contribute in part to longer PFS in our analysis. Besides, we noticed that CNS ORR of the cFAS set was 48.8%, which appeared to be lower than that in the cEFR set. This was mainly due to the high proportion of included patients with non-measurable brain lesions. That’s to say, many cases in the cFAS set cannot be calculated into the ORR, unless the response of patients was assessed as a CR.

The median mTTR was 1.6 months in both cEFR and cFAS sets, indicating a rapid CNS response to afatinib. This comparison supported that afatinib may rapidly shrink the brain metastasis, regardless the tumor size and location, without worrying about radiation necrosis and neurocognitive dysfunctions which may led by brain radiation.

There are few clinical data reporting the CNS activity of afatinib in patients carrying uncommon EGFR mutations. BMs seemed to exert a detrimental influence on the survival of advanced NSCLC patients with G719X/L861Q/S768I (31). Based on a combined analysis of LUX-Lung 3 and 6, CNS ORR was 33.3% (3 of 9) in patients with uncommon EGFR mutations and BMs (23). Outcomes presented by Yang et al. also indicated that afatinib might have encouraging CNS activity against tumors harboring uncommon EGFR mutations (56% in major uncommon mutations, 25% in exon 20 insertions, 9% in T790M and 10% in others) with median CNS TTF of 8.2 months in a subgroup of patients with BMs (32). Our study represents a more comprehensive analysis exploring CNS response to afatinib in patients with BMs harboring uncommon EGFR mutations, as well as comparing the differences of CNS efficacy between the two EGFR mutation cohorts. In our subgroup analysis, the uncommon EGFR mutation cohort consisted of 64.7% (11 of 17) patients with compound mutations and 35.3% (6 of 17) patients with single major uncommon EGFR mutation. We mainly used two statistical methods for the time-to-event analysis to sufficiently evaluate CNS efficacy in both common and uncommon mutation cohorts: CNS PFS and cumulative incidence of CNS failure. We found that afatinib demonstrated pronounced CNS activity in the common EGFR mutation cohort with a significantly superior CNS ORR (cEFR), CNS mPFS and a significantly lower cumulative incidence of CNS failure versus the uncommon EGFR mutation cohort. Nonetheless, the uncommon EGFR mutation cohort also showed favorable outcomes with a CNS ORR of 60% (cEFR) and CNS mPFS of 6.1months. The subgroup analysis provided a preliminary exploration on the activity of afatinib in the uncommon EGFR-mutant NSCLC patients with BMs, and the results showed that afatinib also had encouraging CNS efficacy in patients with uncommon EGFR mutations although inferior to that of common EGFR mutations.

Currently, the first-, second- and third-generation EGFR TKIs are available for EGFR-mutant NSCLC patients with BMs. This inevitably leads to the question of tailing different lines of EGFR TKI treatment to deploy the best whole-course strategy for patients. It’s known that second- and third-generation EGFR TKIs confer superior efficacy over first-generation TKIs in patients with BMs based on a series of clinical trials and retrospective analyses (19, 20, 24–27). However, to date, only limited retrospective analyses have demonstrated clinical efficacy of dacomitinib on BMs since patients with BMs are excluded in Phase III ARCHER 1050 trial. Further prospective studies and real-world analyses are warranted to validate the intracranial efficacy of dacomitinib. Afatinib, another irreversible second-generation EGFR TKI, demonstrates superior survival benefits to first-generation TKIs in EGFR-mutant NSCLC patients with BMs. Data presented in our research also lend support to the use of afatinib as a treatment option for BMs in NSCLC patients with either common EGFR mutations or uncommon EGFR mutations. Due to a stronger ability to cross the BBB and penetrate the CNS, osimertinib, an irreversible third-generation EGFR TKI, has superior CNS activity to first- and second-generation TKIs (33). In FLAURA, osimertinib demonstrated pronounced CNS efficacy with an CNS ORR of 91% in the cEFR set and 66% in the cFAS set, which is superior to first-generation EGFR TKIs, representing a clinically significant treatment option for patients with EGFR mutations and BMs (20). However, there’s a lack of head-to-head clinical trial comparing the CNS efficacy of osimertinib with second-generation EGFR TKIs. The optimal management of targeted therapy for BMs is still unclear. Based on the above, it seems that second- or third-generation TKI is supposed to serve as a prior treatment selection expecting to maximize the efficacy to control brain lesions. Opinions differ from each other when it comes to the selection of second- or third-generation TKIs as first-line treatment. There are pros and cons to both treatment options. It’s reported that T790M accounts for more than half of all cases of acquired resistance to first or second-generation TKIs, but the resistance mechanism of osimertinib remains obscure (34, 35). Based on the subgroup analysis of AURA 3, osimertinib also shows promising CNS efficacy with an CNS ORR of 70% in the cEFR set for patients with BMs and metastatic T790M-positive NSCLC (36). Given the high incidence of acquired T790M-positive status in patients with disease progression following the first- or second-generation TKIs and favorable CNS activity of osimertinib given as a subsequent treatment, sequential use of second-generation TKIs and osimertinib may be potentially a feasible first-choice therapeutic strategy for patients with BMs. In terms of the immature CNS efficacy of dacomitinib, sequential afatinib followed by osimertinib may be a priority, especially in those harboring uncommon mutations. More prospective clinical trials including head-to-head trials are needed to address the question of the optimal management of BMs.

Our study had certain limitations. First, this is a single-center retrospective study that potential for selection bias is inevitable and adverse events data may be under-reported, which may result in slightly inconsistent data compared with other studies. Second, due to the relatively small cohort size of the study, there are limitations to draw firm conclusions on the clinical benefits across different subgroups. A further limitation is that the efficacy of afatinib on leptomeningeal metastases remained unclear as all patients observed in this study had parenchymal but no leptomeningeal metastases. In the next stage, we could conduct a multi-center study as well as expand the sample size to further validate our results and supplement the deficiencies.

Briefly, in this study, afatinib first-line treatment was found to have encouraging efficacy in brain metastases in advanced NSCLC patients harboring either common or major uncommon EGFR mutations in a real-world setting, with manageable toxicities. Patients with common mutations showed better CNS outcomes than those with uncommon mutations.

## Data availability statement

The raw data supporting the conclusions of this article will be made available by the authors. Further inquiries can be directed to the corresponding authors.

## Ethics statement

The studies involving human participants were reviewed and approved by Institutional Review Board of Sun Yat-Sen University Cancer Center and conducted in accordance with the Declaration of Helsinki.

## Author contributions

Conception and design: YBL, YL, LK. Provision of study materials or patients: YBL, YL, WL. Data collection: LK, JM, CH, QZ. Analysis and interpretation of data: LK, JM, WL. Drafting and revision of the manuscript: All authors. Study supervision: YBL, YL. All authors contributed to the article and approved the submitted version.
